# Spondylodiscitis Presenting to a Chiropractor: A Case Report and Literature Review

**DOI:** 10.7759/cureus.35491

**Published:** 2023-02-26

**Authors:** Eric Chun-Pu Chu, Robert J Trager, Sara Jia Mei Goh, John Sing Fai Shum

**Affiliations:** 1 New York Chiropractic & Physiotherapy Centre, New York Medical Group, Kowloon, HKG; 2 Chiropractic, Connor Whole Health, University Hospitals Cleveland Medical Center, Cleveland, USA; 3 Radiology, Hong Kong Advanced Imaging, Kowloon, HKG

**Keywords:** discitis, psoas abscess, epidural abscess, chiropractic, back pain

## Abstract

An 80-year-old man under combination therapy for pulmonary tuberculosis presented to a chiropractor with a one-month history of worsening chronic low back pain, yet denied having any respiratory symptoms, weight loss, or night sweats. Two weeks prior, he saw an orthopedist who ordered lumbar radiographs and magnetic resonance imaging (MRI), showing degenerative changes and subtle findings of spondylodiscitis, but was treated conservatively with a nonsteroidal anti-inflammatory drug. The patient was afebrile, yet considering his older age and worsening symptoms, the chiropractor ordered a repeat MRI with contrast, which revealed more advanced findings of spondylodiscitis, psoas abscesses, and epidural phlegmon, and referred the patient to the emergency department. A biopsy and culture confirmed *Staphylococcus aureus* infection and were negative for *Mycobacterium tuberculosis*. The patient was admitted and treated with intravenous antibiotics. We conducted a literature review revealing nine previously published cases of patients with spinal infection presenting to a chiropractor, who were typically afebrile men with severe low back pain. Chiropractors rarely encounter patients with undiagnosed spinal infections and should manage those suspected of infection with urgency via advanced imaging and/or referral.

## Introduction

Spinal infections may affect the vertebrae, discs, spinal canal, and/or paraspinal soft tissues [1], can progress over a span of weeks, and are potentially fatal [2]. They may be classified as pyogenic (i.e., bacterial), granulomatous (e.g., tuberculosis or fungal), or parasitic [3]. The most common cause is the bacteria *Staphylococcus aureus*, which accounts for 20% to 84% of cases [3]. Spinal infections are rare, affecting between four and 24 individuals per million per year in developed countries [3], yet are more likely to occur among the elderly, immunocompromised, intravenous drug users, and males [3].

The most common presenting symptom of spinal infection is back pain, which is typically constant and worse at night [1,2]. Other symptoms are variable, such as fever, which is found in 48% to 63% of patients [1]. Aside from a lack of consistent, serious, or “red flag” symptoms, degenerative findings on radiography may cloud clinical judgment, further contributing to any diagnostic delay [3]. Given the potentially inconspicuous presentation of spinal infection, such patients may present to chiropractors, who often manage spinal pain [4].

One recent study in Hong Kong found that out of 7,221 patients presenting to chiropractors with a new episode of low back pain, three patients (0.04%) had an undiagnosed spinal infection [4]. Importantly, spinal infections represent a contraindication to common chiropractic treatments such as spinal manipulation [4]. While spinal manipulation is generally safe, it may fracture already weakened bone [5]. Despite the importance of this topic, presentations of spinal infection in the chiropractic office are seldom reported in the literature [4,6,7].

Chiropractors are trained to recognize red flags in the patient’s history and examination that may be suggestive of serious pathology and prompt diagnostic imaging [4]. Examples include age over 70, night pain, worsening despite a trial of conservative therapy, fever, and neurological impairment [8]. In addition, chiropractic education typically includes fundamentals of diagnostic imaging interpretation, with an emphasis on spinal disorders [9]. In several regions, chiropractors are also permitted to order imaging tests [4,9].

Given the limited literature on this topic, we report a patient who presented to a chiropractor with worsening low back pain and was ultimately diagnosed with a pyogenic spinal infection. We also conduct a brief literature review that highlights the role of chiropractors in recognizing and referring such patients.

## Case presentation

Patient information

An 80-year-old male who was actively treated for tuberculosis presented to an integrative chiropractic clinic upon referral from his primary care provider for acute-on-chronic low back pain. He noted a 10-year history of dull, aching, low back pain, which had progressed over the past month to frequent severe pain, currently rated 8 out of 10 on the numeric pain rating scale. Pain radiated into the flank region bilaterally but did not affect the lower extremities. Symptoms were exacerbated by transitional movements and exertion. He denied having any respiratory symptoms, recent weight loss, or night sweats and denied any medical illness aside from tuberculosis and chronic low back pain. However, he did endorse a recent fever and fatigue. He was a retired accountant, non-smoker, and non-drinker, and had no recent surgery or medical procedures. His World Health Organization Quality of Life score was 70%.

Twelve months prior, he developed a cough and was diagnosed with pulmonary tuberculosis via typical findings of pneumonia on chest radiography and a positive tuberculosis blood test (Interferon Gamma Release Assay). His pulmonologist prescribed a regimen of ethambutol hydrochloride (0.25 g), isoniazid (0.1 g), rifampicin (0.15 g), and pyrazinamide (0.25 g), which he was currently taking.

**Figure 1:**
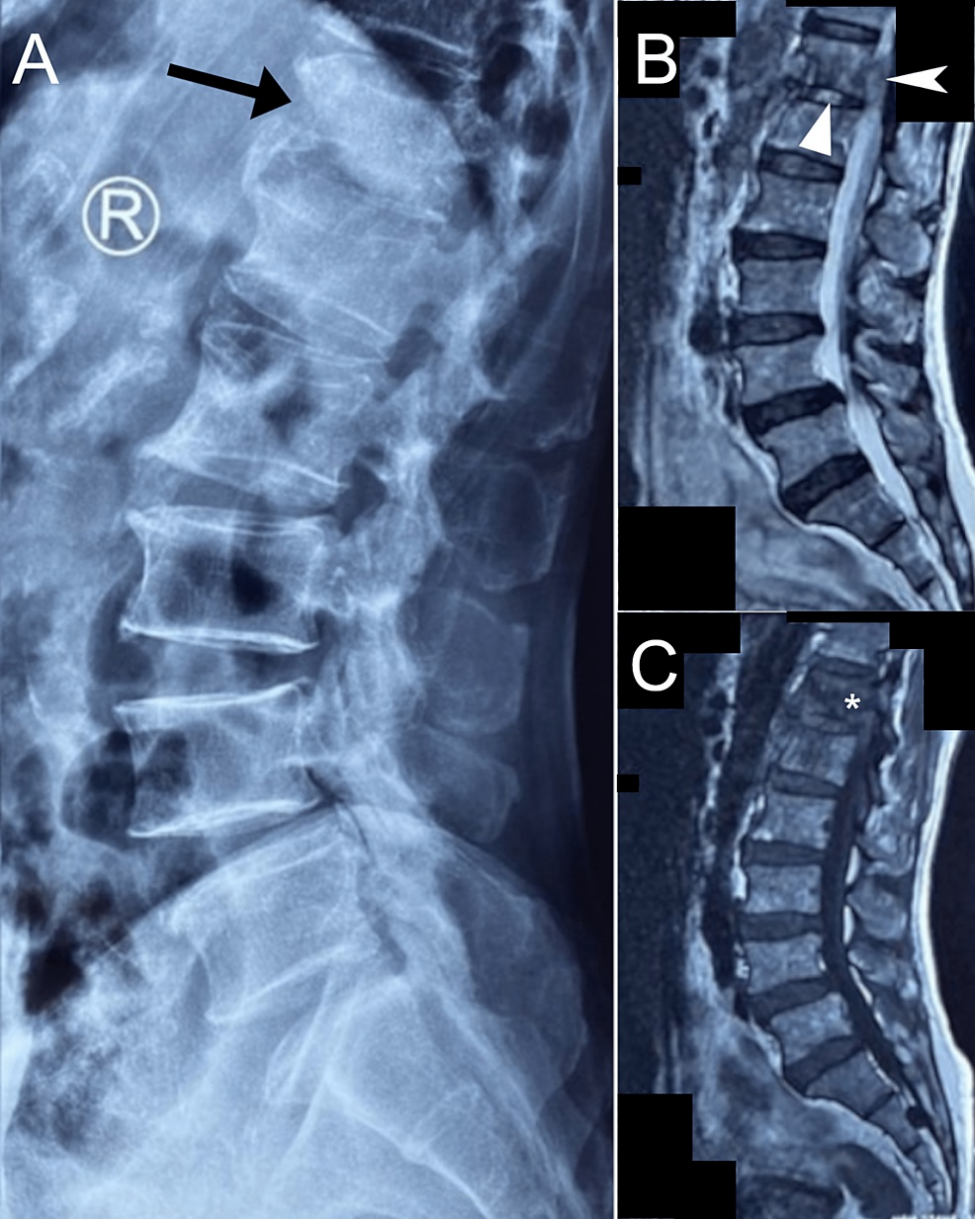
Lumbar spine imaging prior to presentation to the chiropractor Lateral lumbar radiograph (A) and mid-sagittal T2-weighted (B) and T1-weighted (A) magnetic resonance images. On the radiograph, there is an apparent deformity of the T12 vertebral body (arrow). This was re-demonstrated on MRI, which also showed an abnormality in the anterior epidural space (arrowhead) potentially indicative of epidural abscess and was not noted on the original MRI report. Further signs of possible infection were a hyperintense T2-weighted signal of the T12/L1 disc (triangle) and hypointense T12 vertebral body (asterisk*). Image quality was poor as only film could be obtained rather than digital copies.

Sixteen days prior, he visited an orthopedist due to worsening low back pain. The orthopedist performed lumbar radiographs followed by lumbar magnetic resonance imaging (without contrast) two days later. Imaging revealed vertebral body deformity and erosion at T12, a grade I degenerative anterolisthesis of L4 on L5, and a small amount of fluid in the psoas muscles bilaterally, among other changes (Figure 1). While the radiologist suggested spinal infection as a differential diagnosis, the patient was unaware of this as a possibility and was reportedly treated for degenerative low back pain. Per the patient’s records, he was prescribed the nonsteroidal anti-inflammatory drug celecoxib (0.2 g), advised to remain active but avoid carrying heavy objects, and return to the orthopedist if needed. The patient did not have a complete blood count, any inflammatory marker tests, blood culture, or biopsy at this time. Given the patient’s worsening symptoms, he contacted his primary care provider who referred him to a chiropractor.

Clinical information

Upon presentation to the chiropractic clinic, the patient was observed to be very thin. He had a forward antalgic posture, appearing to relate to a bilateral hip flexion contracture, and ambulated slowly, using a cane. He required assistance to transition from sitting to standing. His active lumbar flexion and extension were severely limited and painful. Palpation of T12/L1 and the quadratus lumborum bilaterally was moderately painful. A slump test created a pulling sensation, perceived in the posterolateral thigh, when performed on either side. Hip flexion was limited with 3/5 strength bilaterally (Medical Research Strength Council scale). Other lower extremity motor, sensory, and reflex tests were normal.

Considering the patient’s red flags, including his advanced age, progressive symptoms despite conservative treatment, and previous MRI abnormalities, the chiropractor considered a differential diagnosis of spondylodiscitis, compression fracture, malignancy, and disc herniation. The chiropractor urgently ordered a lumbar MRI with gadolinium contrast to better evaluate the patient’s possible spinal infection, which the patient obtained on the same day of presentation.

**Figure 2:**
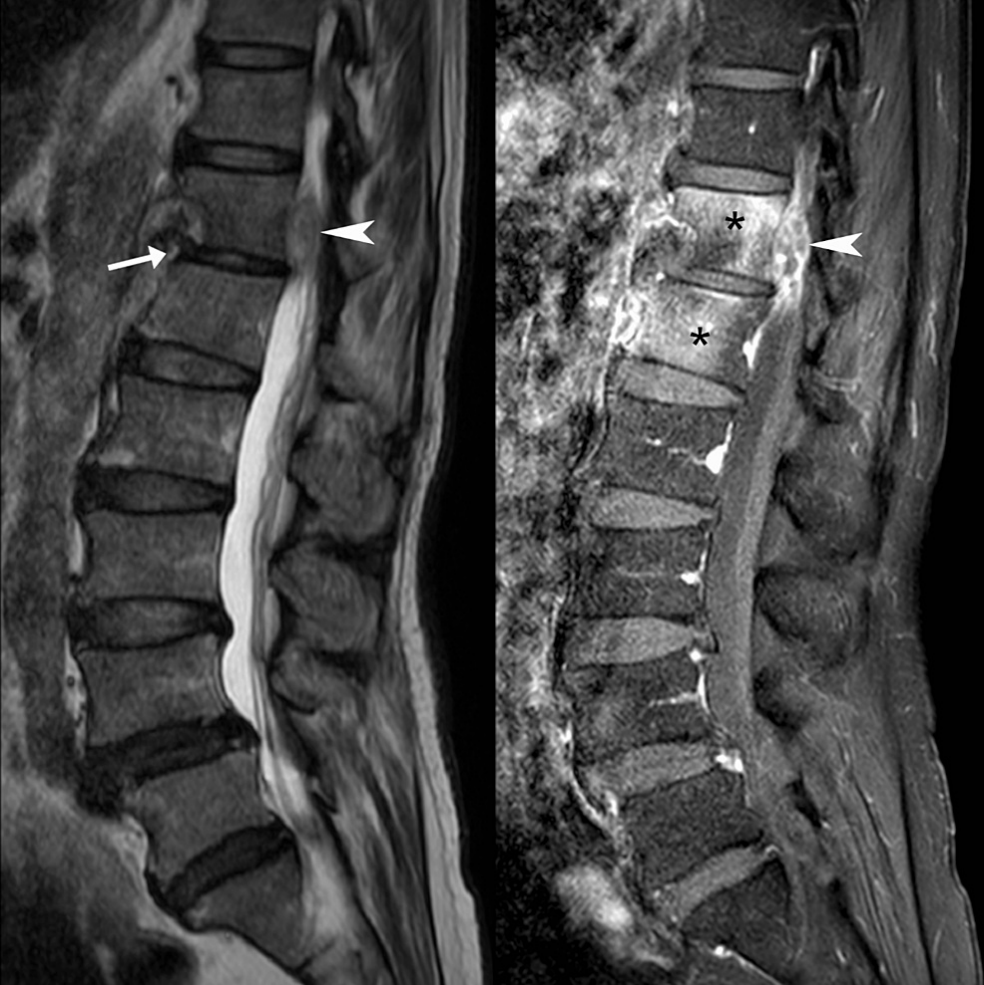
T2-weighted (A) and T1-weighted post-contrast (B) sagittal lumbar magnetic resonance imaging Several signs of pyogenic spinal infection were evident, including a reduction in the T12/L1 disc space (arrow), heterogeneously contrast-enhancing anterior epidural fluid collection, consistent with an epidural phlegmon (arrowhead), and contrast enhancement of the T12 and L1 vertebral bodies (asterisks *), consistent with vertebral osteomyelitis.

**Figure 3:**
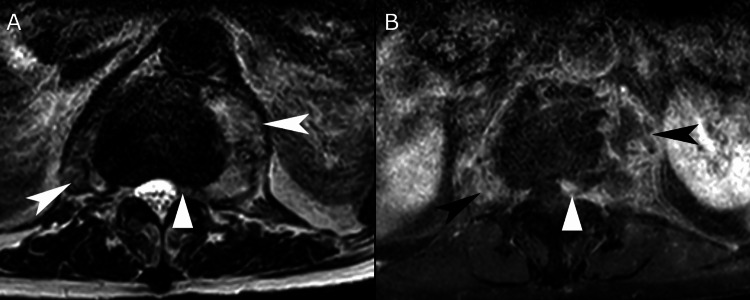
T1-weighted (A) and T1-weighted post-contrast (B) axial lumbar magnetic resonance imaging at the level of the T12/L1 intervertebral disc A rim contrast-enhancing psoas abscess with a thick and irregular wall is evident bilaterally, larger on the left (arrowheads), which extends into the epidural space (triangle).

The MRI revealed interval worsening since the MRI two weeks prior, including bilateral psoas abscesses, epidural phlegmon, and spondylodiscitis, which was suggestive of pyogenic spinal infection (Figure 2, Figure 3). The radiologist alerted the chiropractor of the urgency of these findings. Accordingly, the chiropractor consulted with the clinic’s on-staff neurosurgeon and was advised to immediately refer the patient to the emergency department.

The patient’s family brought him to the hospital, where he was admitted. Psoas abscess biopsy with culture confirmed S. aureus spinal infection and was negative for M. tuberculosis and other pathogens (e.g., Actinomyces, Brucella, Burkholderia, Candida, Histoplasma). Reportedly, the patient was also advised neurosurgical treatment, yet declined, and was administered intravenous antibiotics. The patient provided written consent for publication of this case report but was later lost to follow-up.

## Discussion

This case highlights an elderly man with acute-on-chronic worsening low back pain who was identified as having a pyogenic spinal infection after visiting a chiropractor. The patient was afebrile upon presentation; however, it is possible that his celecoxib normalized his temperature since he did report a recent history of fever. The patient did have a history of tuberculosis; however, he had been on combination therapy for nearly a year. Completion of a 12-month course of antibiotics is associated with a high success rate of curing tuberculosis (85-89%) [10]. Further, relapses typically occur after the therapy is completed [10], whereas our patient was still actively taking medication.

The patient’s low back pain was multifactorial and unfortunately may have created a confusing clinical picture. He had chronic low back pain and degenerative changes on imaging, notably at L4/5. In addition, his previous provider focused on these findings when proposing a treatment plan. Clinicians should realize that degenerative findings are common, especially in the elderly population [11]. In the current case, long-standing degenerative changes would not likely explain the patient’s acutely worsening features. In addition, this case highlights that subtle imaging signs of spinal infection should be managed with urgency.

Tuberculous and pyogenic spondylodiscitis were the key differential diagnoses in the current case. Fungal or parasitic causes of spondylodiscitis are much less common; the patient had no specific risk factors for these infections (e.g., immunocompromise, endemic area), and the imaging features were inconsistent with these etiologies (e.g., fungal spondylodiscitis generally lacks contrast enhancement) [2,3]. However, tuberculous and pyogenic spondylodiscitis may be difficult to distinguish based on their clinical and imaging features [12]. This was especially true in the current case, as the patient had a history of tuberculosis, yet had imaging features consistent with pyogenic infection. Regardless, the patient’s older age was a risk factor for spinal infection in general. The biopsy and culture were confirmatory of *S. aureus,* which represents the most common etiologic agent of spinal infection [3].

In the current case, several imaging features were characteristic of pyogenic infection including (1) epidural phlegmon (with heterogenous contrast enhancement), (2) hypointense vertebral body on T1-weighted sequences, (3) intervertebral disc involvement and narrowing, notably hypointense on T1-weighted sequences, (4) involvement of only two spinal levels, (5) absence of involvement of the posterior elements of the spine, and (6) a thick and irregular psoas abscess wall [12,13]. In comparison, tuberculous spondylodiscitis tends to involve more than two vertebral levels, and classically involves the subligamentous spread of disease, with pronounced destruction of anterior vertebral elements and subsequent loss of anterior vertebral body height [12,13]. These latter features were absent in our patient. As evident in the current case, contrast agents enhance the diagnostic accuracy of MRI in spinal infection [13].

We searched the literature for similar cases describing patients with an undiagnosed spinal infection presenting to a chiropractor, which included a complete case history, excluding gray literature. We queried PubMed, Google Scholar, and the Index to Chiropractic Literature on February 14, 2023, using variations of the terms “chiropractor,” “spondylodiscitis,” “epidural abscess,” “psoas abscess,” “spinal infection,” and “vertebral osteomyelitis,” hand-searched a recent review article [14], and performed citation tracking of included articles.

This search identified nine cases (Table 1) [4,6,7,15-18]. Including the current case, there were 10 patients, with a mean age of 55±12, a symptom duration of 7±15 weeks, and 90% of patients were male. The most common presenting symptom was low back pain, occurring in 70% of patients. In cases reporting pain severity (n=6), it was always severe (i.e., ≥7 out of 10). Among cases reporting the patient’s temperature (n=6), none reported a fever, although in the current case and one other [16], both patients reported a recent subjective fever. Red flags and/or risk factors were variable, with patients having diabetes (n=2), night pain (n=1), bilateral radiculopathy (n=1), human immunodeficiency virus (n=1), or active smoking (n=1).

**Table 1:** Summary of cases of undiagnosed spinal infection presenting to chiropractors Abbreviations: Female (F), human immunodeficiency virus (HIV), low back pain (LBP), male (M), not reported (NR)

Author, year	Patient age, sex	Symptoms & duration	Red flags or risk factors	Fever on presentation	Diagnosis	Management
Chu, 2022 [4]	50, M	LBP, 1 week	Night pain	NR	Psoas abscess, spondylodiscitis	Pharmacotherapy
Chu, 2022 [4]	60, M	LBP with lower extremity symptoms, 1 week	Bilateral radiculopathy	NR	Epidural abscess	Pharmacotherapy
Chu, 2022 [4]	60, F	LBP & hip pain, 1 week	None	NR	Vertebral osteomyelitis	Pharmacotherapy
Chu, 2022 [19]	60, M	Severe LBP with radiation bilaterally, neck pain, 2 weeks	Poorly controlled diabetes	No	Paraspinal, psoas, and epidural abscesses (pyogenic)	Intravenous antibiotics
Chu, 2023 (current case)	80, M	Severe LBP with radiation to flank, difficulty walking, 1 month	Age >70, worsening despite treatment, recent fever	No	Pyogenic spondylodiscitis, psoas abscesses, epidural abscess/phlegmon	Intravenous antibiotics
Cupler, 2017 [7]	59, M	Severe thoracic spine pain, 8 days	Smoking	No	Pyogenic spondylodiscitis, epidural abscess, & phlegmon	Laminectomy of T7-8, fusion of T6-9, and antibiotics
Fogeltanz, 2006 [15]	44, M	Severe neck pain, headaches, 2 weeks	Night pain, constant pain	No	Retropharyngeal abscess	C1/2 fusion, antibiotics
Kanga, 2015 [18]	32, M	Severe LBP with radiation to thigh, 11 months	HIV	NR	Tuberculous spondylodiscitis	Pharmacotherapy
Kim, 2004 [17]	50, M	LBP with radiation to the leg, 6 weeks	None	No	Pyogenic spondylodiscitis	Intravenous antibiotics
Murphy, 2006 [16]	52, M	Severe neck pain, 1 week	Recent fever, diabetes mellitus	No	Epidural abscess	Patient passed away

This case has several limitations. As a previous provider may have overlooked subtle, but important MRI abnormalities, the diagnosis of pyogenic spondylodiscitis could potentially have been made sooner. Early diagnosis of this condition is critical to reducing morbidity and mortality [2]. It was unclear what the patient’s source of *S. aureus *infection was, as the patient had no recent known surgery, wounds, or apparent cellulitis. After we referred the patient to an outside hospital for emergent treatment of his spinal infection, we had limited clinical information regarding his response to treatment. In addition, we could not obtain further records regarding laboratory or imaging tests. According to a recent survey of Asia-Pacific chiropractors (n=241), only 23% reported working in an integrative setting with medical physicians [20], as in the current case. Therefore, chiropractors in private practice settings may not have the opportunity to consult with an on-staff radiologist and neurosurgeon. In addition, chiropractors in some regions of the world may not be able to order diagnostic imaging. Such chiropractors may thus opt to refer patients with suspected spinal infections sooner.

## Conclusions

We highlight an older man with worsening low back pain diagnosed as pyogenic spondylodiscitis after visiting a chiropractor. Our literature review identified nine similar patients with a spinal infection presenting to a chiropractor, who were typically afebrile men with severe low back pain. Although fever may not be present on examination, chiropractors should be able to recognize other red flags and risk factors for spinal infection (e.g., night pain, immune compromise, diabetes, male sex, and older age). For patients suspected of having a spinal infection, chiropractors should urgently obtain advanced imaging and/or refer them for medical management.
